# Effect of Two-Step Sintering on Properties of Alumina Ceramics Containing Waste Alumina Powder

**DOI:** 10.3390/ma15217840

**Published:** 2022-11-07

**Authors:** Milan Vukšić, Irena Žmak, Lidija Ćurković, Andraž Kocjan

**Affiliations:** 1 Faculty of Mechanical Engineering and Naval Architecture, University of Zagreb, Ivana Lučića 5, 10000 Zagreb, Croatia; 2 Department for Nanostructured Materials, Jožef Stefan Institute, Jamova cesta 39, 1000 Ljubljana, Slovenia

**Keywords:** waste alumina, recycling, two-step sintering, mechanical properties, microstructure

## Abstract

This study aims to evaluate the recycling potential of solid waste alumina powder (WAP) by utilization of the two-step sintering (TSS) process. For the study, WAP was collected as an industrial scrap after the machining process for the formation of green alumina compacts. The alumina samples were prepared according to the slip casting method by preparing suspensions containing commercial alumina with 0.8 μm average particle size and by adding up to 20 dwb. % (i.e., expressed on a dry weight basis) of WAP with 3.4 μm average particle size. The samples were sintered at optimized TSS conditions and compared with conventional one-step sintering (OSS) by conducting morphological analyses. The average grain size (AGS) was determined from the obtained field emission scanning electron microscopy (FESEM) images, while the sample porosity was calculated based on apparent densities. The obtained micrographs after TSS implementation revealed a partially textured microstructure. Furthermore, a comparison of the mechanical properties of alumina samples lacking or containing 20 dwb. % of WAP obtained after sintering is presented. The indentation fracture toughness (~3.2 MPa m^1/2^) and Vickers hardness data (~14.5 GPa) showed a positive effect of adding WAP to alumina samples. The slightly improved mechanical properties of ceramic samples containing waste alumina are a consequence of lower porosity, which is due to the remaining sintering additives in WAP. The collected results demonstrate the possibility of using TSS for sintering ceramic materials that contain WAP.

## 1. Introduction

The modern manufacturing processes are focused on the reduction or elimination of waste generation inside the production line to achieve cleaner production and consequently protect the environment. It is necessary to safely store the waste generated during the manufacturing process according to valid regulations [1]. In some cases, a more cost-effective manner is to recycle generated waste as secondary raw material for the new production cycle. This approach can lead to cost reductions due to omitting the costs related to storage and landfill deposition of generated waste [2,3]. Additionally, the implementation of recycled material can result in the refinement of material properties [4]. Ceramic industry, with a wide range of products, is an adequate candidate for improving waste management by implementing the internal recycling of generated waste. This refers especially to alumina-based engineering ceramic materials, which are one of the most widely used in industrial production [5].

In our previous study [6], the possibility of recycling waste alumina powder (WAP), which is generated during the green machining step of alumina green bodies, was reported. The prepared samples containing different amounts of WAP were obtained via conventional sintering and compared with pure alumina samples. The addition of WAP positively affected the obtained microstructure, which demonstrated average grain size refinement from ~3 μm to ~2 μm. The addition of WAP increased bulk densities as well, thus improving the mechanical properties. In the present study, the beneficial contribution of two-step sintering (TSS), a non-conventional sintering method, on the microstructural evolution was investigated to evaluate the possibility of additional improvement of the mechanical properties for alumina ceramics containing up to 20 dwb. (dry weight basis) % WAP.

The TSS is a cost-effective process, and it can be easily applied in a sintering furnace at lower temperatures compared to conventional sintering [7]. As the name suggests, the sintering process consists of two steps. In the first step, the sample is heated (usually up to 10 °C min^−1^) to the temperature which will achieve the relative sample density > 75% of the theoretical density (TD). Then, the temperature is rapidly lowered to the point where the sintering process is continued for a substantially longer period of time (usually a couple of hours) [8,9]. The sintering at lower temperature results in the densification of the ceramics by suppressing the grain growth.

The described process was successfully used for obtaining smaller grain size of alumina ceramics compared to the conventional one-step sintering (OSS) [10,11]. In the reported studies [12,13], submicron-sized alumina powders with a narrow-sized distribution were mostly used. The used TSS regimes resulted in refined microstructure with finer grains compared to the conventional sintering process. At the same time, lower theoretical densities were achieved due to the defects present in the prepared green bodies [14].

In this study alumina samples containing 20 dwb. % of WAP and without WAP addition were prepared by the slip casting technique. The prepared green bodies containing WAP were sintered via TSS according to the developed Box–Behnken experimental design (BBD), which was used to determine favorable sintering conditions. The favorable conditions, i.e., the temperature of the second sintering stage, heating rate, and the holding time were established, based on achieved density values as the monitored response. The grain sizes of two types of alumina samples sintered at optimized conditions were determined. Furthermore, samples containing 20 dwb. % of WAP and pure (commercial) alumina samples were sintered at favorable TSS conditions. Afterwards, the samples were compared to conventional one-step sintering (OSS) in terms of grain size and mechanical behavior, namely Vickers hardness and indentation fracture toughness.

## 2. Materials and Methods

### 2.1. Green Body Preparation

The chemical composition of used alumina powders is provided in Table 1. The high-purity (99.9% α-Al_2_O_3_, Alcan Chemicals, Stamford, CT, USA) alumina powder with average particle size from 0.4 μm to 1.2 μm and WAP with average particle size from 2.32 μm to 4.37 μm were used for the green body sample preparation. The WAP was collected after machining of green compacts during the industrial manufacturing process of engineering alumina ceramics, as described in our previous work [15].

The weight loss of as-received alumina powders (commercial and WAP) was analyzed by the thermogravimetric method (TG-DTA/DSC instrument STA 449 C/6/G Jupiter–QMS 403, Netzsch, Selb, Germany). The TG-DTA method was carried out by applying a heating rate of 10 °C min^−1^ up to 1400 °C under atmospheric conditions.

The green bodies were prepared by the slip casting technique, which involves the preparation of a stable highly concentrated ceramic aqueous suspension. The water-based 70 wt. % alumina suspensions were produced by using 0.05 dwb. % of dispersant Tiron^®^ (Sigma-Aldrich Chemie GmbH, Munich, Germany), 0.1 dwb. % of binder polyvinyl alcohol (Sigma-Aldrich Chemie GmbH, Munich, Germany), and finally 0.2 dwb. % of magnesium aluminate spinel (Alfa Aesar GmbH, Haverhill, MA, USA) as a sintering additive. The alumina suspensions containing WAP were prepared by the addition of 20 dwb. % of WAP, while samples without the addition of WAP were prepared in the same way as those with WAP. A detailed explanation of suspension composition and stability investigation is reported in previous work [16]. To obtain ceramic green bodies, the prepared suspensions were cast into prefabricated gypsum molds with an inner diameter of 21 mm. After demolding, the cube-shaped green bodies were obtained and were dried at ambient temperature for 24 h. The geometric green density was determined by calculating the ratio between mass and volume of the dried green bodies. The mass was weighed by the digital analytical libra (Ohaus AP250D, Ohaus Europe GmbH, Zurich, Switzerland), and the volume was calculated from measured geometrical dimensions of the green bodies using Holex^®^ micrometer, Hoffmann Group, Munich, Germany.

### 2.2. Box–Behnken Experimental Design and Statistical Analysis

The response surface methodology (RSM) is comprised of various statistical and mathematical methods which can be practically used for developing and optimizing various processes. RSM is often used in industry to determine the influence of different input variables on the quality properties of the developed product or manufacturing process. Usually, the measured quality properties of the monitored process are known as the response, while the investigated input variables are also known as process or independent variables. The response surface model is obtained by approximating the polynomial equation to the obtained experimental data [17]. For the purposes of this study, the Box–Behnken design tool was selected to determine the TSS conditions for the production of ceramic materials which contain WAP.

The reduced second-order model was used as an approximation to generate a response surface. In general, the second-order polynomial model can be presented by Equation (1) as follows [17]:

(1) $$Y=\beta_{0}+\overset{k}{\underset{i=1}{\sum }}\beta_{i}x_{i}+\overset{k}{\underset{i=1}{\sum }}\beta_{ii}x_{i}^{2}+\sum \overset{k}{\underset{i<j=2}{\sum }}\beta_{ij}x_{i}x_{j}$$

where *Y* represents the measured response variable, for example the apparent density of the sintered ceramic samples, while *x_i_* and *x_j_* represent the independent variables, and finally *β*_0_, *β_i_*, *β_ii_*, *β_ij_* represent the regression coefficients. The model fitting to experimental data and checking the adequacy of a second-order model was performed by Design-Expert^®^ software (ver. 11.1.2). Furthermore, analysis of variance (ANOVA) was used to identify the effects of independent variables on monitored response, the significance of the developed model, and interactions between model terms. The coefficient of multiple determination *R*^2^ was calculated to check the fitness of the developed second-order model. The statistical significance of the developed model and model terms was determined by the probability (*p*-value) approach, which is commonly used for statistical testing (*F*-test). The statistical testing was performed at a *p*-value of 0.05 to establish the favorable TSS conditions. Finally, the favorable sintering conditions were verified by conducting three additional runs.

### 2.3. Two-Step Sintering (TSS) of Green Bodies

The TSS of ceramic green bodies was conducted in an electric furnace (LH 04/18, Nabertherm GmbH, Lilienthal, Germany) in an air atmosphere. The temperature of the first sintering step, *T*_1_, of 1550 °C was achieved by applying different heating rates ranging from 4 to 10 °C min^−1^ followed by a holding time, *t*_1_, of 5 min. The temperature of 1550 °C, in the first step, was chosen in order to fulfil the condition by which >75% of theoretical density for the sintered material needs to be achieved. The time duration of the first sintering step was based on previously reported studies [18,19]. After setting up fixed sintering parameters, the remaining sintering parameters were investigated in selected range according to the literature [11,18,19] by using Design-Expert^®^ software (Stat-Ease, Inc., Minneapolis, MN, USA) to determine favorable sintering conditions. The Box–Behnken response surface design was developed by including three parameters of interest in the following ranges: temperature of the second sintering step, *T*_2_, from 1300 to 1400 °C; heating rate from 4 to 10 °C min^−1^; and holding time of the second sintering step, *t*_2_, from 2 to 6 h. The cooling rates between temperatures *T*_1_ and *T*_2_ and the cooling to room temperature after isothermal holding at *T*_2_ were not controlled. The developed experimental design resulted in a total of 15 experimental runs, including three repetitions of the central point (Table 2). 

At least five samples were simultaneously sintered per experimental run. The linear shrinkage was measured by studying the thermal behavior of the alumina sample during sintering at favorable conditions in a vertical optical dilatometer (Misura^®^ ODHT, Expert System Solutions S.r.l., Modena, Italy). The favorable conditions were determined based on the monitored response of bulk density achieved after sintering. Additionally, conventional one-step sintering was conducted for comparison by applying a sintering temperature of 1650 °C, heating rate of 4 °C min^−1^, and holding time of 5 h.

### 2.4. Microstructural and Mechanical Characterization

The apparent density of sintered samples was determined according to the Archimedes principle by using a density meter (JP703C, Mettler-Toledo GmbH, Greifensee, Switzerland) using water as immersion medium. The theoretical density of 3.980 g cm^−3^ was taken as a referent value for the relative density calculations of sintered alumina samples [20]. Furthermore, the alumina samples containing and lacking WAP sintered at optimal conditions were characterized by microstructural analysis. The samples were ground by a Struers Rotopol-11, Struers, USA, machine to obtain the polished surface. Firstly, the diamond plates from larger to finer granulation (MD-Piano 220, MD-Piano 1200, MD-Largo) were used, followed by additions of 9 μm, and finally 3 μm, diamond paste on MD-Dac plate. Afterwards, ceramic samples were thermally etched and coated with gold to obtain micrographs through field emission scanning electron microscopy (FESEM) analysis (FEI Helios Nanolab 650, Thermo Fisher, Waltham, MA, USA). The line intercept method [21] was performed when measuring at least 500 grains/per sample to determine average grain sizes (AVGs).

The hardness values were determined according to ASTM C1327-15(2019) by the Vickers method (*HV*10), which was performed by applying a load of 98.1 N in the indentation time of 10 s at least 5 times per sample using a hardness tester (Innovatest, Nexus 7500, Maastricht, The Netherlands). The dimensions of the corresponding indentations and subsequent crack lengths were processed by an optical microscope (Carl Zeiss, Axio Imager Z1m, Oberkochen, Germany). The obtained lengths were also used to determine fracture toughness (*K*_IC_). Numerous mathematical models were proposed for the determination of fracture toughness by using the Vickers indentation fracture (IF) method [22,23,24]. The developed mathematical models enable the calculation of fracture toughness (*K*_IC_) by relating to cracks formed in the material during hardness testing as given by Equation (2):

(2) $$K_{\mathrm{IC}}=\beta_{0}\;\times \;{\left(E/H\right)}^{1/2}\;\times \;\left({P/c}^{3/2}\right)$$

where *β*_0_ is the parameter which value depends on the proposed mathematical model, *E* is the elasticity modulus, *H* is the measured Vickers hardness of the material, *P* is the applied load during the hardness testing, and *c* is the average crack length measured from the center of the indentation imprint [25]. In this research, the fracture toughness of the sintered alumina ceramics samples was computed using Evans and Charles’s [26] Equation (3) as well as Tanaka’s [27] Equation (4):

(3) $$K_{\mathrm{IC}}=0.0752\;\times \;\left({P/c}^{3/2}\right)$$

(4) $$K_{\mathrm{IC}}=0.0725\;\times \;\left({P/c}^{3/2}\right)$$

## 3. Results and Discussion

### 3.1. Thermal Analysis of Alumina Powders

**Figure 1:**
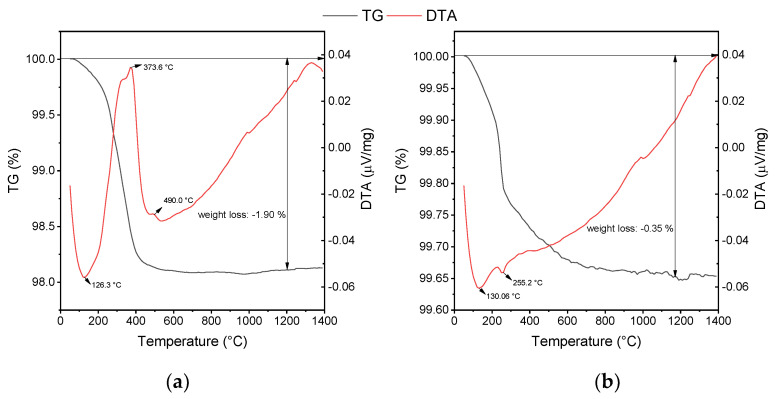
DTA/TG curves of: (**a**) waste alumina powder and (**b**) pure alumina powder.

Thermogravimetry/differential thermal analysis (TG/DTA) is the most frequently used method of thermal analysis for determining the organic and inorganic content of various materials. Figure 1 illustrates the effect of increasing temperature on the weight loss of the as-received WAP and commercial alumina powder. All weight changes happened during heating cycle until 1400 °C were reached. The total weight loss of the WAP was around 2 wt. % as a result of water evaporation close to 130 °C and thermal degradation of present organic compounds with increasing temperature (Figure 1a). More precisely, the weight loss was observed at 373.6 °C and 490 °C due to the thermal decomposition of organic additives [28]. A further increase in temperature did not show any weight loss of the WAP. Negligible weight loss of commercial alumina powder was recorded at 130 °C due to a small amount of moisture, and some hydroxides at 255 °C [29] (Figure 1b). Additionally, a slight decrease in weight above of 500 °C could be contributed to transient alumina dihydroxylation [30,31]. The small moisture content of 0.35 wt. % is attributed to the hygroscopic nature of the ceramic powder.

**Figure 2:**
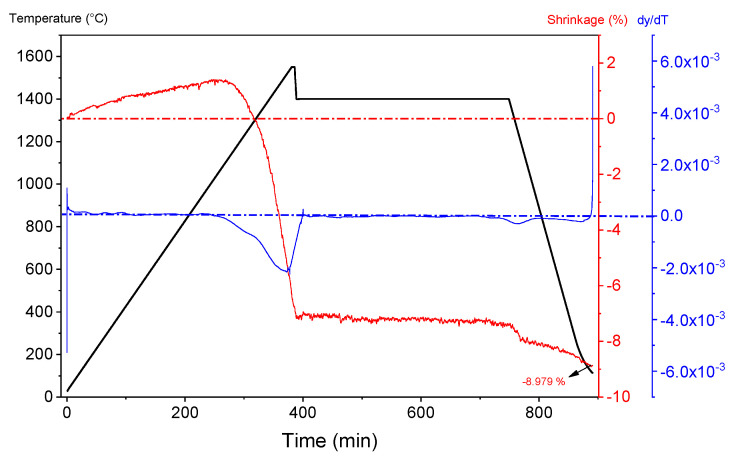
Dilatometry analysis of alumina containing 20 dwb. % of WAP sintered at optimal TSS conditions.

Dilatometric curves were recorded for alumina containing 20 dwb. % of WAP, sintered at favorable TSS conditions (Figure 2). The onset sintering, i.e., densification temperature of about 900 °C is visible. The derivative curve is bimodal, demonstrating firstly a slower shrinkage, followed by a more pronounced one. The first shrinkage could be related to continued decomposition of some hydroxides (Figure 1b) with the increase in temperature, hence the transition from *δ* to *θ* to *α*-alumina, which occurs between 1050 and 1200 °C [32,33,34]. The pronounced shrinkage was attributed to the annihilation of pores on the account of grain boundary diffusion at lower temperatures of the second sintering step [35]. A total linear shrinkage of about 9% was determined.

### 3.2. Determination of Two-Step Sintering Conditions

The apparent density which was achieved for different TSS conditions was the monitored response (Table 3). Additionally, the variation of the observed responses with the change of independent variables (TSS conditions) is presented. The experimental values of the apparent density were fitted to the polynomial second-order model to obtain the most adequate regression equations. The predicted responses were calculated from the following regression equation:

(5) $${Y\;(\mathrm{density},\;g\;\mathrm{cm}}^{-3})=2.93768+0.000646\;\times \;X_{1}+0.075958\;\times \;X_{2}+0.008292\;\times \;X_{3}\;\;-5.33\;\times \;10^{-5}\;\times \;X_{1}\;\times \;X_{2}\;-\;0.000792\;\times \;X_{2}\;\times \;X_{3}$$

where:

- X_1_ is the temperature of the second sintering step (°C),
- X_2_ is the heating rate (°C min^−1^),
- X_3_ is the holding time (h).

The presented regression Equation (5) describes the relationship between the independent variables and the response, which is the apparent density of the sintered samples at various TSS conditions.

The significance, goodness of fit, and the coefficients of determination for the developed model were determined by the analysis of variance (ANOVA), as presented in Table 4.

The *F*-test and the associated *p*-values (*p* < 0.05) were determined for the developed model [36,37]. The *F*-value of 20.45 and the appropriate *p* = 0.001 suggest that the reduced quadratic model of the density response is a significant model. Furthermore, the determined *F*-value (0.4473) for the model lack of fit was insignificant (*p* >> 0.05), which validates the developed model. The satisfactory values for the coefficient of determination *R*^2^ = 0.9191 and the adjusted coefficient of determination $R_{\mathrm{Adj}.}^{2}$ = 0.8742 were determined. The values do not differ substantially, which is a consequence of excluding the nonsignificant quadratic terms from the developed model. The obtained coefficients of determination indicate that the reduced form of the developed model can be used to describe the relationship between the monitored apparent density and applied TSS conditions. The coefficient of variation of the model (C.V. = 0.1183%) strongly suggests a high precision of the experimental values. Additionally, the adequate precision ratio was 15.025, which suggests that the developed model can be applied to the entire design area.

The significance of model terms from Equation (5) indicates (Table 4) the significant terms (*p* < 0.05) are X_1_ and X_3_, and the quadratic interaction X_1_X_2_. Other model terms showed less effect (X_2_X_3_, *p* < 0.1) or an insignificant effect (X_2_, *p* > 0.1) on the apparent density.

### 3.3. Interactive Effects of TSS Conditions on Apparent Density

**Figure 3:**
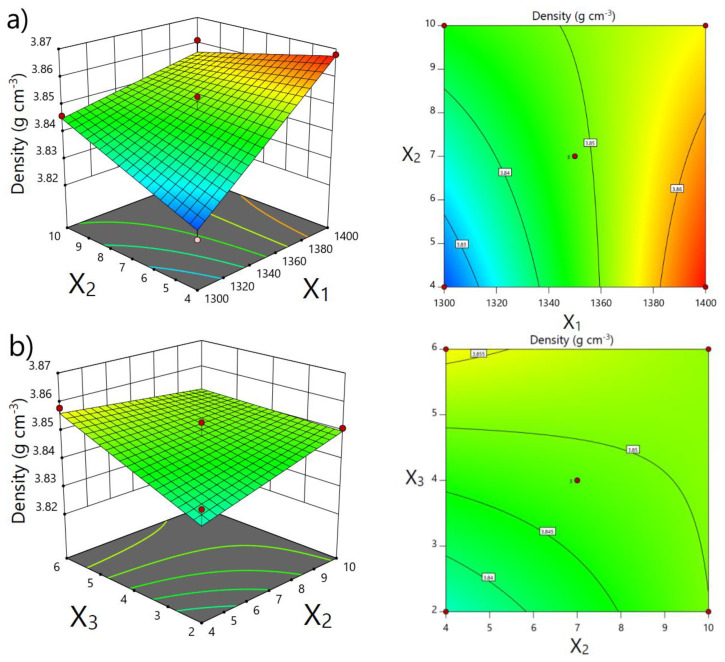
3D response surface and contour plots depicting influence of: (**a**) sintering temperature of second step (X_1_) and heating rate (X_2_) and (**b**) holding time (X_3_) and heating rate (X_2_) on apparent density.

The effects of the temperature of the second sintering step, heating rate, and holding time on the apparent density of sintered samples were depicted using 3D response surface and contour plots (Figure 3a,b). The plots were generated by keeping constant one variable at center level (0), while the remaining two variables were continuous in the investigated range to visualize their effect on the apparent density.

The temperature of the second sintering step showed the most significant effect on the increase of the apparent density of the sintered samples. The density values increased by increasing temperature *T*_2_ from 1300 °C to 1400 °C. The third independent variable, X_3_, the holding time at temperature *T*_2_, also had a significant effect on the obtained densities. It can be clearly seen that the achieved density is higher with increasing holding time, although the incline in the apparent density in the plots is not as obvious as in the plot for temperature *T*_2_. The change in heating rates in the investigated range did not affect density values.

The favorable conditions of TSS, determined to obtain the maximal apparent density of the sintered samples, are as follows: the temperature of the second sintering step of 1400 °C, the heating rate of 4 °C min^−1^, and holding time of 5 h. The holding time of 5 h was determined as favorable because the difference in the achieved density between applied holding times of 5 and 6 h is negligible. Therefore, from an economic point of view, it is justified to apply a holding time of 5 h (Figure 3b). The validation of the predicted favorable conditions was conducted by sintering at least three samples. The obtained density for TSS at determined favorable conditions was 3.862 g cm^−3^ ± 0.007 g cm^−3^ (~97 % TD) for samples containing 20 dwb. % of WAP, and 3.820 g cm^−3^ ± 0.020 g cm^−3^ (~96 % TD) for samples lacking WAP, respectively. Both densities are in good agreement with the predicted value of 3.869 g cm^−3^ ± 0.006 g cm^−3^. The reported density of alumina achieved by TSS varies from to 96 % TD [19] to 99 % TD [11].

### 3.4. Microstructure of Sintered Alumina

**Figure 4:**
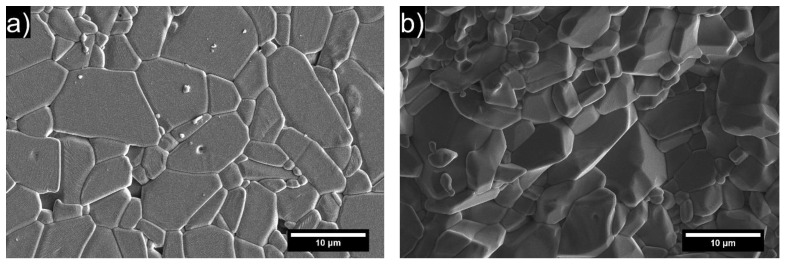

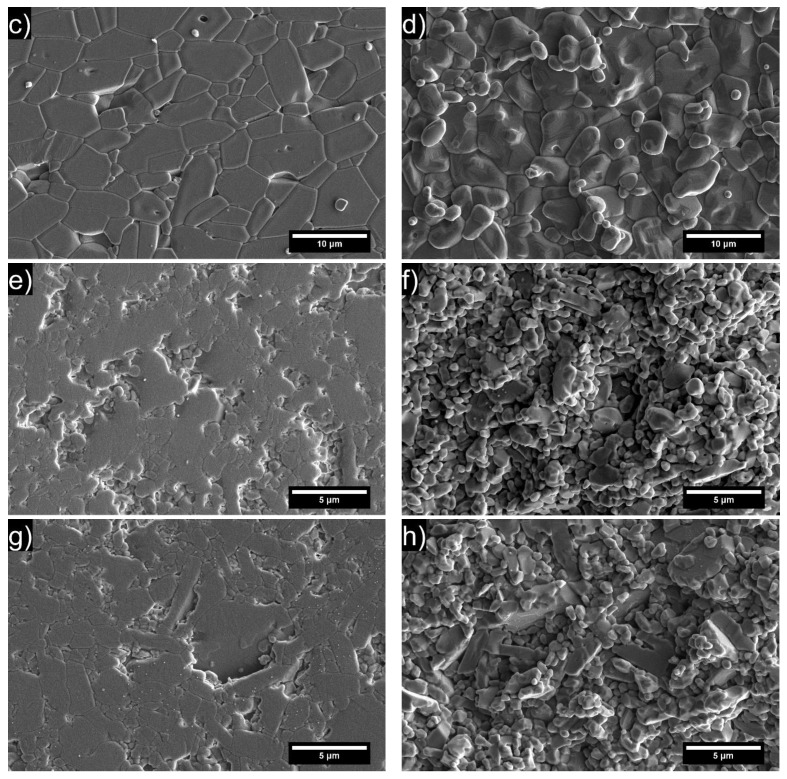
FESEM images of alumina surface microstructure. (**a**) OSS/0 dwb. % WAP; (**c**) OSS/20 dwb. % WAP; (**e**) TSS/0 dwb. % WAP; (**g**) TSS/20 dwb. % WAP and of respective sample fracture surface: (**b**) OSS/0 dwb. % WAP; (**d**) OSS/20 dwb. % WAP; (**f**) TSS/0 dwb. % WAP; (**h**) TSS/20 dwb. % WAP.

The obtained FESEM micrographs of samples sintered by TSS at favorable conditions were compared with micrographs obtained by conventional OSS (Figure 4). The presented microstructures are relatively dense, while sporadic intergranular pores were observed. The addition of WAP has positively affected the obtained microstructures, resulting in slightly increased density values and reduced average grain size, as presented in Table 5. The organic binders present in the WAP (Figure 1) contributed to higher relative/green densities of alumina samples with the addition of WAP.

**Figure 5:**
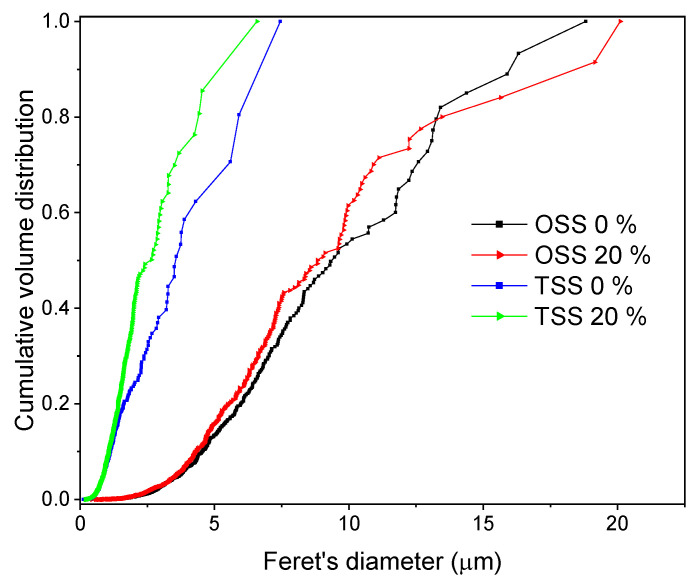
Cumulative volume grain size distribution versus Feret’s diameter of sintered samples with different waste alumina content.

The microstructures with substantially smaller grains can be noticed for samples sintered by TSS regardless of WAP content. This is clearly presented in Figure 5, with all data listed in Table 5. In addition to the reduced grain growth, larger oriented plate-like particles served as templates for the generation of similarly oriented grains during the sintering process, as can be observed from the elongated grains in TSS micrographs (Figure 4e,g). The thermal gradients contributed to texturing via preferential growth of aligned grains, thus resulting in partially textured ceramics [38,39]. Furthermore, micrographs showing fracture surfaces of TSS samples exhibit crack propagation along boundaries of grain aggregates 3–5 μm in size formed due to favorable TSS mode. The “embossed” fracture surface comprising cleavage facets of elongated grains is evidence of high cohesion between grains merged into aggregates and, as a result, comparatively high fracture toughness of the TSS/ 20 dwb. % WAP material. (Figure 4f,h). The fracture surface micrographs of OSS samples revealed transgranular crack propagation without any visible crack deflection (Figure 4b,d).

The cumulative grain size distribution (GSD) for each composition shows that the narrowest distributions were achieved when TSS was used for sintering of alumina samples (Figure 5). The microstructures obtained by this sintering procedure showed a finer average grain distribution with partial consolidation (higher porosity) of the microstructure (Figure 4, Table 5). The widest distribution of grain sizes was determined for the microstructure obtained by the OSS process with a higher degree of consolidation. From the presented cumulative volume distribution curves, the positive effect of the addition of WAP, which contributed to even further narrowing the grain size distribution (GSD) of alumina samples sintered by the TSS process, can be seen. The effect of the addition of WAP on GSD of samples sintered by the OSS process was less obvious but was still present. Furthermore, it was determined that 80 vol. % of grains sintered by TSS had a diameter smaller than 5.91 μm and 4.42 μm, for samples lacking WAP and containing 20 dwb. % of WAP, respectively. The curves of GSD for samples sintered by OSS demonstrated larger grains compared to TSS, with 80 vol. % of grains smaller than 13.25 μm for the samples with the addition of WAP, and 13.45 μm for samples without the addition of WAP.

The determined Vickers hardness (*HV*10) were ~14.7 GPa for OSS 0 and ~14.5 GPa for OSS 20. Lower values of Vickers hardness, ~10.2 GPa for TSS 0 and ~12.1 GPa for TSS 20, were obtained due to higher porosity of TSS samples compared to OSS samples. Lower densities obtained by TSS resulted in lower hardness values compared to conventionally sintered samples, although a significant reduction of average grain size was achieved (Table 5). The applied conventional sintering method with a high sintering temperature of 1650 °C boosted the consolidation rate, which led to an increase of AGS with more pronounced grain boundaries. It was expected that the addition of the WAP would result in finer microstructures due to the increased MgO content (Table 1). The stronger effect of WAP addition is less noticeable due to magnesium spinel, which was used as a sintering additive during the preparation of alumina green bodies.

**Figure 6:**
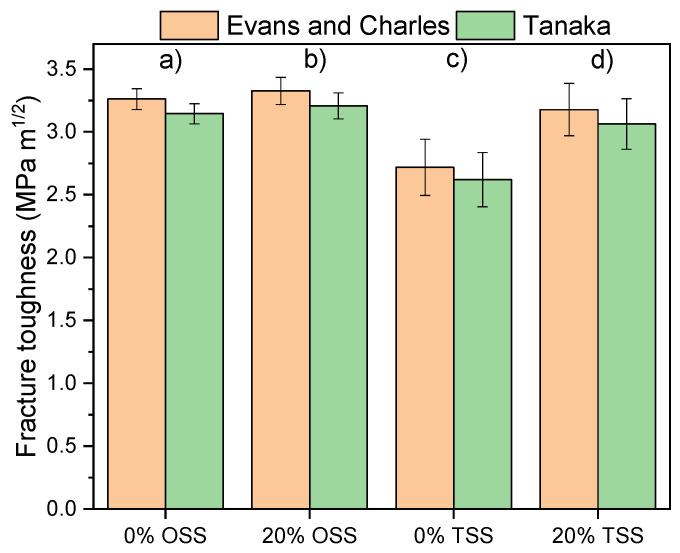
The calculated fracture toughness according to median crack models for: (a) OSS/0 dwb. % WAP; (b) OSS/20 dwb. % WAP; (c) TSS/0 dwb. % WAP; (d) TSS/20 dwb. % WAP.

The indentation toughness (*K*_IC_) values were computed according to Evans/Charles [26] and Tanaka [27] median crack models (Figure 6). A slight increase in fracture toughness with the addition of WAP was registered when OSS was used. A larger increase in fracture toughness was observed when the samples containing WAP were sintered using TSS. The lowest values in fracture toughness were observed for samples TSS 0, which had the highest porosity. The samples TSS 20 are on par with OSS samples despite the higher porosity of TSS 20 samples. This can be explained by promoted crack deflection due to textured microstructure (Figure 4e–h), which subsequently increased fracture toughness of TSS 20 samples [40]. In general, all calculated *K*_IC_ values were in the range from 2.7 MPa m^1/2^ to 3.5 MPa m^1/2^, which is in agreement with the literature values for alumina engineering ceramics [41,42].

## 4. Conclusions

The favorable conditions for TSS of alumina samples containing up to 20 dwb. % of WAP were determined. The TSS conditions of *T*_1_ = 1550 °C, *t*_1_ = 5 min, *T*_2_ = 1400 °C, *t*_2_ = 5 h with the applied heating rate of 4 °C min^−1^ were used to achieve the maximal apparent density in the investigated range. The obtained sintered samples were compared with samples without WAP and with samples sintered by conventional OSS. The significant sintering parameters were the temperature of the second sintering step (*T*_2_) and holding time at *T*_2_ (*t*_2_), while the change in heating rate was insignificant in the investigated range. The achieved relative densities (~97%) were lower compared to OSS (~98%) because of lower *T*_2_, which is necessary to avoid grain growth. In addition, lower relative densities were obtained for alumina samples without WAP due to sintering additives present in the waste alumina powder. Generally, regardless of WAP content, the samples sintered by TSS demonstrate reduced average grain size of around 1 μm with noticeable elongation of grains (texturing) and higher porosity compared to the conventionally sintered samples with AGS of around 4 μm. This resulted in decreased hardness values (~10.2 GPa for TSS 0 and ~12.1 GPa for TSS 20) and in slightly changed indentation fracture toughness values of ~3.2 MPa m^1/2^ for TSS 20, as compared to the conventionally sintered samples. The addition of WAP did not result in degradation of mechanical properties, which suggests the feasibility of recycling WAP as secondary raw material in ceramic manufacturing. However, some limitations for alumina-containing WAP may be noted. In particular, the limited use of such material can be in applications where strong requirements are placed on the color or other surface features of ceramic products, as the resulting microstructure may still cause the effects of changing the color of the product and other factors.

## Figures and Tables

**Table 1:** Chemical composition of pure and waste alumina powder.

Powder	Component	MgO	Fe_2_O_3_	SiO_2_	Na_2_O	CaO	Al_2_O_3_
Pure	wt. %	0.06	0.02	0.02	0.05	0.01	rest
WAP ^1^	wt. %	0.10	0.02	0.02	0.08	0.03	rest

^1^ Obtained after green machining step from industrial facility for alumina ceramics manufacturing.

**Table 2:** Box–Behnken experimental design for the three levels (−1, 0, and 1) of factor X_1_ (temperature of the second sintering step), X_2_ (heating rate), X_3_ (holding time).

Run	Temperature of Second Sintering Step (°C)		Heating Rate (°C min^−1^)		Holding Time (h)	
	Coded	Actual	Coded	Actual	Coded	Actual
1	0	1350	−1	4	1	6
2	1	1400	0	7	1	6
3	0	1350	1	10	−1	2
4	0	1350	0	7	0	4
5	1	1400	1	10	0	4
6	−1	1300	−1	4	0	4
7	1	1400	0	7	−1	2
8	0	1350	1	10	1	6
9	1	1400	−1	4	0	4
10	−1	1300	1	10	0	4
11	0	1350	0	7	0	4
12	0	1350	0	7	0	4
13	0	1350	−1	4	−1	2
14	−1	1300	0	7	−1	2
15	−1	1300	0	7	1	6

**Table 3:** Monitored responses and predicted data of applied BBD.

Box–Behnken Design					
Run	X_1_ Temperature of Second Sintering Step	X_2_ Heating Rate	X_3_ Holding Time	Y Apparent Density	
	(°C)	(°C min^−1^)	(h)	Experimental (g cm^−3^)	Predicted (g cm^−3^)
1	1350	4	6	3.858	3.856
2	1400	7	6	3.865	3.867
3	1350	10	2	3.851	3.850
4	1350	7	4	3.853	3.848
5	1400	10	4	3.861	3.856
6	1300	4	4	3.821	3.824
7	1400	7	2	3.852	3.856
8	1350	10	6	3.849	3.851
9	1400	4	4	3.868	3.868
10	1300	10	4	3.846	3.845
11	1350	7	4	3.841	3.848
12	1350	7	4	3.848	3.848
13	1350	4	2	3.841	3.836
14	1300	7	2	3.827	3.829
15	1300	7	6	3.843	3.840

**Table 4:** ANOVA results for fitted regression model of monitored response.

Source	Apparent Density Model			
	Sum of Squares	df	*F*-Value	*p*-Value
**Model**	0.0021	5	20.45	0.0001
**X_1_**	0.0015	1	71.69	<0.0001
**X_2_**	0.0000	1	2.18	0.1741
**X_3_**	0.0002	1	30.83	0.0077
**X_1_X_2_**	0.0003	1	2.66	0.0066
**X_2_X_3_**	0.0001	1	3.75	0.0665
**Residual**	0.0002	9		
**Lack of fit**	0.0001	7	0.4473	0.8225
**Pure error**	0.0001	2	$R_{}^{2}$ = 0.9191	
**Total**	0.0023	14	$R_{\mathrm{Adj}.}^{2}$ = 0.8742	
**C.V. % = 0.1183**		**Adequate precision** = 15.0250		

**Table 5:** Properties of alumina ceramics sintered conventionally and at determined favorable TSS conditions.

Sample ID	Sintering Method	WAP (dwb. %)	*T*_1_ (°C)	*T*_2_ (°C)	*t*_2_ (h)	Green Density (%)	Relative Density (%)	AGS (µm)	*HV*10 (GPa)
OSS 0	OSS	0	1650	-	5	59.43 ± 2.31	98.20 ± 0.53	3.91 ± 2.65	14.70 ± 0.45
OSS 20	OSS	20	1650	-	5	62.77 ± 2.14	98.40 ± 0.30	3.63 ± 2.49	14.48 ± 0.30
TSS 0	TSS	0	1550	1400	5	61.01 ± 7.59	95.98 ± 0.50	0.87 ± 0.66	10.18 ± 0.34
TSS 20	TSS	20	1550	1400	5	57.67 ± 3.75	97.04 ± 0.18	0.88 ± 0.60	12.10 ± 0.44

## Data Availability

The authors declare that all data supporting the findings of this study are available within the article.
